# Education level has an effect on the recovery of total knee arthroplasty: a retrospective study

**DOI:** 10.1186/s12891-022-05939-w

**Published:** 2022-12-07

**Authors:** Yuan-yuan Zhou, Bo-kai Zhang, Tian-fei Ran, Song Ke, Tian-ying Ma, Yin-yin Qin, Yuan Zhang, Yuan Xu, Min Wang

**Affiliations:** 1 Department of Orthopaedics, the Second Affiliated Hospital of Army Medical University, Xinqiao Street, Chongqing, 400037 Shapingba China; 2 Department of Orthopaedics, Third Military Medical University), Xinqiao Hospital, Amy Medical University, Xinqiao Street, Chongqing, 400037 Shapingba China

**Keywords:** Total knee replacement, Education level, Rehabilitation, ROM

## Abstract

**Objective:**

This study aimed to observe the relationship between education level and outcomes after total knee arthroplasty (TKA).

**Methods:**

One thousand two hundred sixty four patients after TKA in our hospital from April 2016 to April 2020 were reviewed. These patients were divided into 4 groups (A who were illiterate, B who had elementary school degree, C who had junior high school degree, D who had senior high school degree or higher) by the educational level, which was blinded to the observers. The postoperative outcomes of KSS score, pain, joint extension and flexion function were observed 1 year after discharged from hospital.

**Results:**

Among 1253 patients met the inclusion criteria, the average age was 68.63 years, the average body mass was 57.73 kg. There are no distinctions among 4 groups one day after the surgery. However, the outcomes of the follow up were that, the KSS score was: 77.84 ± 10.635; 80.70 ± 8.956; 87.92 ± 8.123;91.27 ± 8.262, with significant differences (*P* < 0.05). The mean VAS scores were: 1.97 ± 1.60; 2.07 ± 1.66; 1.197 ± 1.5265, 1.044 ± 1.4662. Patients in Group C and D had significantly less pain than that in Group A and B (*P* < 0.05). The knee flexion range of motion (ROM) was: 91.21 ± 11.69°; 91.77 ± 11.95°; 102.12 ± 11.38°; 109.96 ± 10.64°, Group D performed best, with significant differences (*P* < 0.05). The knee extension ROM were: – 2.41 ± 4.49°; – 0.91 ± 2.82°; – 0.83 ± 2.87°; – 0.35 ± 1.60°, with significant difference between Group D and the others (*P* < 0.05).

**Conclusion:**

Education level affects the outcomes such as VAS score, KSS score, the extension and flexion ROM of the knee after TKA. The patients with higher education level have better outcomes.

## Introduction

As the society developing and the living standard of people rising, it is undeniable to confirm that the number of people suffering from primary osteoarthritis, rheumatoid arthritis, traumatic arthritis, gout who desires to get a total knee arthroplasty(TKA) is getting increasingly numerous, with an estimate that the number of total knee arthroplasty cases will surge from 711 000 in 2011 to 3.48 million by 2030 in the United States, not to mention in China [1, 2]. However, the prognosis doesn’t satisfy every patient [3]. At present, there are still 20% of patients who are not satisfied with the knee after the total knee arthroplasty [3, 4]. The main grumbles of the patients after osteoarthritis are mainly about the pains and the restricted range of motion [5] (ROM). The limitation in function accompanied by increasing pain will eventually affect the patient’s well-being which is not only social but emotional, which doesn’t meet the primary propose of total knee arthroplasty by alleviating pain and majorization physical function and quality of life [6, 7].

The recovery exercise after the surgery is commonly viewed as an indispensable factor in improving post-operative outcomes such as pain and flexible function [8]. However, the insufficient postoperative rehabilitation training of patients is one of the reasons contribute to the unsatisfaction [9]. Patients are often discharged 1–3 days after surgery, in China [10]. In such a short hospital stay time patients were mainly educated on rehabilitation training by doctors and rehabilitation therapists, while the rehabilitation was usually performed and acted by patients themselves at home [11]. So how much content the patients would obtain from the rehabilitation training will be differing, to some degree it may depend on the education level. Thus function recovery (pains and ROM) after total knee arthroplasty are distinctive [5].

The elements that affect the comprehension of recovery education of total knee arthroplasty include the patients` body conditions, education levels (EL), social experience, and so on [11]. It has been shown in some studies that education level which affects the compliance of patients is an independent factor for every patients’ efficiency of rehabilitation practices after total knee arthroplasty [6, 12]. However, definitive evidence on relevant studies and classification of education level is lacking, which resulted in the conclusions uncertain at present [6]. We guessed that the rehabilitation of total knee arthroplasty might be different in different education level groups, and the batter education classification could lead to batter recovery. So we stared this study in order to discover evidence-based conclusion of the effect of education level on outcomes improvement after total knee arthroplasty by assess different education levels of patients whose function recovery ranges from patients who are illiterate to patients who enter to university. Fortunately, we came into a conclusion. When culture levels vary, the outcomes of patients getting rehabilitation education vary, through differing the compliance to the recovery exercise of the after total knee arthroplasty patients, which would provide as a relatively reliable data for physical therapists who make the Rehabilitation Programs.

## Materials and methods

### Patients

#### Inclusion criteria

The inclusion criteria were: i) patients who had been confirmedly diagnosed with primary unilateral or bilateral knee disorders such as osteoarthritis, ii) rheumatoid arthritis, gout iii) and traumatic arthritis. iv) patients who had undergone unilateral and first total knee arthroplasty; v) patients who could be retrospectively recruited.

#### Exclusion criteria

The exclusion criteria were: i) patients who had undergone periprosthetic joint infection (PJI), ii)patients who had been suffering from ankylosis, Charcot joint and extra-articular deformity, or iii)patients who had tumor, iv)unicondylar replacement; or v) patients who had vascular claudication, vi)history of major trauma, diabetic polyneuropathy; vii) or patients who had undergone total knee arthroplasty already because of tibial plateau fracture or the patients with problems of surgical error. viii) or patients who couldn’t bear the pains got the painkillers such as morphine. Patients who met any of the exclusion criteria must be excluded from the study.

All the 1264 patients in this study who were invited to be the subjects were gotten primary total knee arthroplasty in our hospital from April 2016 to April 2020 and were retrospectively reviewed, including 239 males (18.91%), 1025 females (81.09%), the mean values were 68.63 ± 8.00 years for age (range: 34–92) and 25.93 for body mass index (BMI) (range: 20–34), the middle body mass was 67.73 ± 14.44 kg. And the stature mean values were 158 ± 8.20 cm.

This study was a retrospective study. All subjects received and accepted informed consent before participating in the study. This study was approved by the institutional review committee of our hospital (Reference number: 2018-YD-084–01).

### Diagnosis

Knee joint diseases were diagnosed by three experienced joint surgeons who had already registered professional physician certificate, all with 25-year-experence about osteoarthritis, rheumatoid arthritis, traumatic arthritis, gout, through imaging examination including X-ray, computed tomography (CT), magnetic resonance imaging (MRI) combined with the current symptoms.

### Surgery

All surgical procedures were performed by three experienced surgeons who had 25 years experiences of total knee arthroplasty. The surgical method is standard parapatellar medial approach for total knee arthroplasty. Tourniquet was intraoperatively applied no more than 60 min.

### Intervention

Preoperative long-standing X-rays radiography was performed in all patients in order to achieve a neutral mechanical axis alignment postoperatively. All patients get the recommendation to participate the preoperative patient education session, which was given and supported by two physiotherapist, one anesthesiologist, one orthopedic surgeon, and one nurse. The session lasts approximately 1 h, including relieves tension caused by the surgery, the notice before and after the anesthesia, as well as the postoperative care.

The treatment included analgesic management, all routine subarachnoid anaesthesia, which was operated by 3 experience anesthetists who had qualification already with 10-year-work of anesthesia, postoperative anticoagulation (with Rivaroxaban) and anemia management followed ERAS requirements [13, 14] and based on specific conditions of patients. Patients ambulated on Day 2–3 after surgery and meanwhile practiced auxiliary exercise of continuous passive motion (CPM). The target of the motion was to get rid of postoperative adhesion of the tissue concerned. This protocol consisted of range-of-motion in knee, 5°-10°, activities with the help from the family or nursing assistance [15]. On the second day after surgery, the drainage was removed, standing-position X-ray of knee joint was taken, and two rehabilitation physiotherapists guided the knee rehabilitation.

### Clinical evaluation

According to the highest educational level certificates they got which were provided by the patients or their families, these patients were divided into 4 groups: illiterate group (Group A), elementary school group (Group B), junior high school group (Group C) and senior high school above group (including senior high school and university) (Group D).

#### American Knee Society knee score (KSS 2011)

American Knee Society knee score (KSS 2011) was used to access the postoperative outcomes from two parts by a scale. One part is to record the affected side knee’s degree of pain, which ranges from 0 to 50, and 0 for insupportable pain 50 for no pain, and knee function, which includes the flexion and extension range of motion, the stability of the knee. The flexion and extension were measured by using a long-arm protractor, and every 5° equaled 1 point. The anterior and posterior motion was measured by a long-arm protractor and ruler. For anterior motion, no more the 5 mm got 10. 5-10 mm got 5, and more than 10 mm got 0. For posterior motion, no more than 5° got 15. 6°-9°got 10, and 10°-14° got 5. Those who moved more than 15° got 0. The second part is the capability to walk both on flat road and upstairs. Who couldn’t walk at all scored 0, and those who could only walk in the room got 10, and 20 for no more than 500 m outside, 30 for 500–1000 m, 40 for more than 1000 m. 50 for who couldn’t be restricted. Who couldn’t go upstairs at all got 0 and those who could go upstairs by the armrest but couldn’t go downstairs got 15, and 30 for those who could go upstairs and downstairs by the armrest,40 for who could go upstairs but need an armrest to go downstairs got, 50 for no restricted [13, 14]. The total KSS score ranges from 0 to 200.

#### Postoperative pain (VAS score)

VAS was used to evaluate the pain of the low back pre- and post- operative. It ranges from 0 to 10, where 0 represents no pain and 10 the worst possible pain.

#### Knee extension function and flexion function

Knee extension function, and flexion function data were assessed and analyzed respectively, and the extension and flexion range of motion (ROM) were measured by using a long-arm protractor when the patient was lying flat. Patients with bilateral total knee arthroplasty were measured in the more painful side. This study was approved by the Ethics Committee of Xinqiao Hospital, Army Medical University. (Reference number: 2018-YD-084–01).

### Data collection

The average hospital stay was 6 days. the follow-up was conducted 1 year after discharge by rehabilitation physiotherapists. The clinical data were obtained through outpatient follow-up, telephone consultation and video follow-up (by Wechat), which was token by three nurses who were experienced enough with five years working time. And they were blinded to the grouping information of the patients. For patients who had communication difficulties, their clinical data were obtained through consultation with close relatives. Those who had bilateral total arthroplasty would be measured two sides and got the mean value. The mean follow-up was 3.65 years (range: 1 to 5 years).

### Statistical analysis

A SPSS 20.0 (IBM, New York, USA) statistical software was used for the analysis of all data, chi-square test or Fisher's exact test were used for analyzing enumeration data, and t-test was used for inter-group comparison of quantitative data. The significance level is α = 0.05 with two-sided values taken, the 95% confidence interval was used, *P* < 0.05 was deemed as significantly different. What’s more, after significantly difference was seen between the groups, Hedges' g was calculated. Hedges' g, which provides a measure of effect size weighted according to the relative size of each sample, is an alternative where there are different sample sizes. It was used to explain the effect size of each comparation. And g > 0.15 indicted small effect size, g > 0.4 indicted medium, and g > 0.8 interpret large effect size [16].

## Results

### Basic patient data

There were 794 unilateral-knee-replacement cases and 470 bilateral-knee-replacement cases in the all 1264 patients. However, postoperative acute PJI occurred in 5 patients (2 patients in Group A, 2 patients in Group C, and 1 patient in Group D), and delayed PJI in 3 patients (1 patient each in Groups A, B, and C), periprosthetic fracture occurred in 3 patients (2 patients in Group A and 1 patient in Group B), after excluding these patients, there were 1,256 cases for analysis: 296 cases in Group A (23.57%), 614 cases in Group B (48.89%), 231 cases in Group C (18.39%), and 115 cases in Group D (9.15%). Fortunately, there was no case of COVID-19. during the pandemic of COVID-19 the patient who had had total knee arthroplasty spent most of their time at home. There was no significant difference in gender and age between the groups, which were comparable. All patients were followed up in outpatient or by smartphone. Moreover, the KSS of each group, one day before they discharged from the hospital, were 50.85 ± 9.625, 50.79 ± 10.542, 50.83 ± 9.935, 50.84 ± 9.733. There was no significant difference among the four groups. The VASs were 9.1 ± 1.59, 9.36 ± 1.78, 9.18 ± 1.45, 9.40 ± 1.55. The extension ROMs were 5.16 ± 1.59, 5.16 ± 1.78, 5.18 ± 1.45, 5.40 ± 1.55. The flexion ROMs were 54.21 ± 11.55˚, 53.47˚ ± 10.36˚, 55.28 ± 9.95˚, 54.43 ± 10.65˚. And there was no significant difference in the index above among the four groups one day before they discharged from the hospital.

### Clinical results

#### American Knee Society knee score (KSS 2011)

**Fig. 1:**
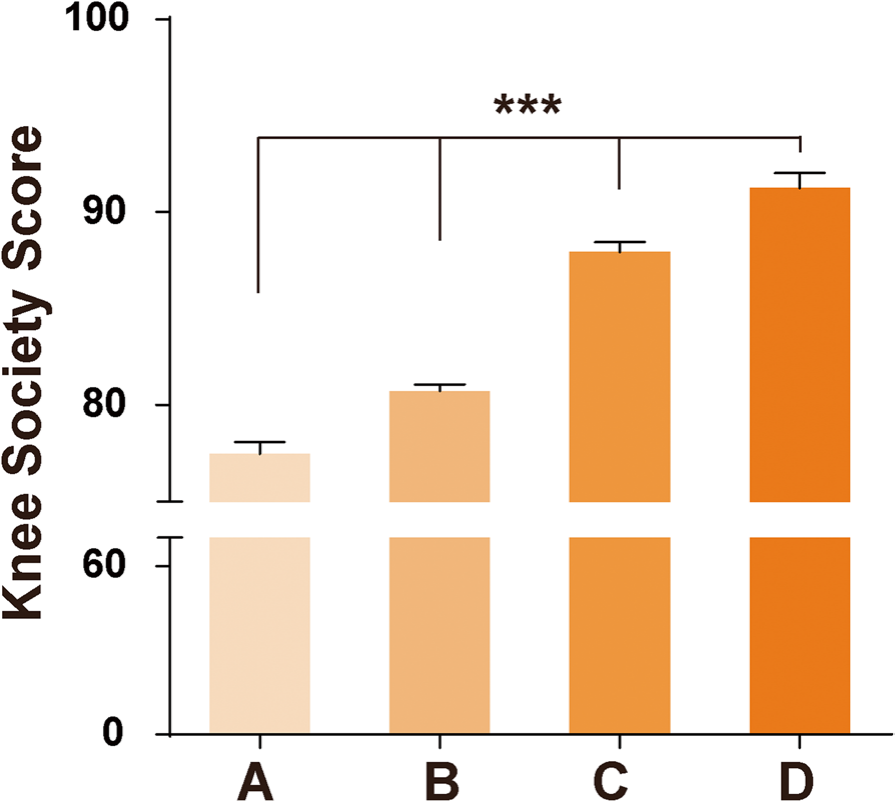
KSS score of each group. There were significant differences when group A was compared with Groups B, C and D (*P* < 0.05)

The average KSS scores of all groups reached good levels and above, with scores of 77.84 ± 10.635 in Group A; 80.70 ± 8.956 and 87.92 ± 8.123 in Group B and Group C, respectively; and 91.27 ± 8.262 in Group D; thedifferences between the groups were significant (Fig. 1). The Hedges' g in group AB, AC, AD: 0.3, 1.048, 1.339. The Hedges' g in group BC, BD, CD: 0.826, 1.194, 0.41. The effect sizes were large in comparation AC, AD, BC, BD(g > 0.8). However, it was medium in comparation of group C and D (g > 0.4), and small in group A and B (g > 0.15). (Table 1) There were 25 patients with scores less than 60 points, including 9 cases in Group A, 11 cases in Group B, 4 cases in Group C and 1 case in Group D, accounting for 3.04%, 1.79%, 1.73% and 0.87% of each group. Respectively, the proportion in Group A was the highest, while the proportion in Group D was the lowest. All the patients could walk without walking aids 3 months after surgery, and some elderly patients had weak walking and needed carrying walking aids when going out.

**Table 1:** Effect size of each comparations

Group comparation	KSS score	VAS score	Flexion ROM	Extension ROM
A-B	0.3	/	/	0.434
A-C	1.048	0.493	0.944	0.409
A-D	1.339	0.592	1.644	0.527
B-C	0.826	0.537	0.877	/
B-D	1.194	0.629	1.547	0.21
C-D	0.41	/	0.704	0.19

In KSS score, the effect sizes were large in comparation AC, AD, BC, BD (g > 0.8). It was medium in comparation of group C and D (g > 0.4). In VAS score: the effect sizes were medium in comparation AC, AD, BC, BD (g > 0.4). In Flexion ROM: the effect sizes were large in comparation AC, AD, BC, BD (g > 0.8). It was medium in comparation CD (g > 0.4). The effect sizes were medium in comparation AB, AC, AD (g > 0.4). It was small in comparation BD, CD (g > 0.15)

#### Postoperative pain (VAS score)

**Fig. 2:**
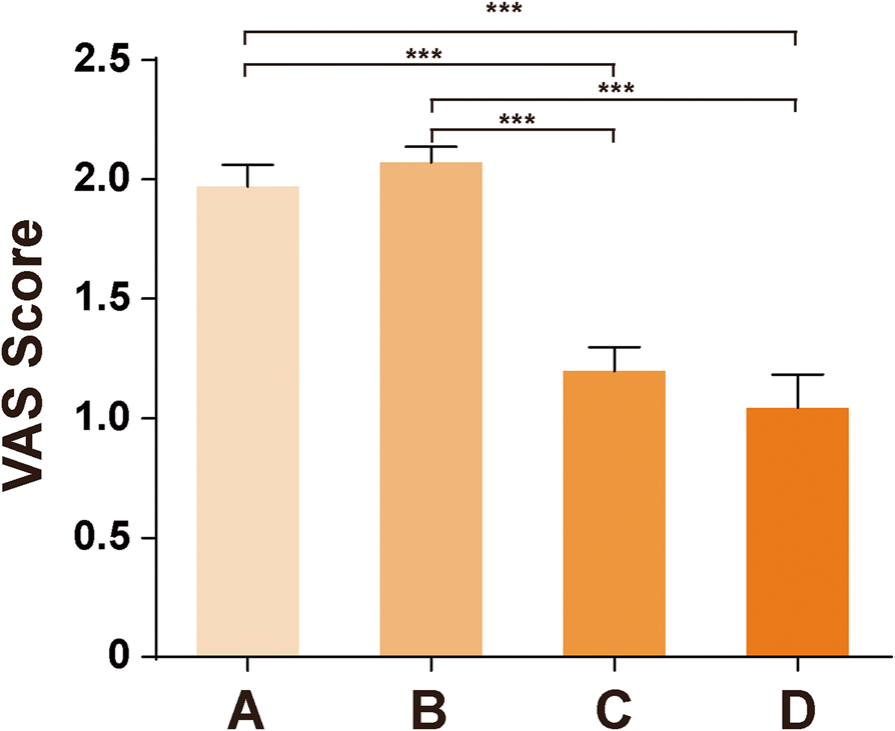
VAS score of each group. There was no significant difference between Group A and Group B (*P* = 0.37 > 0.05) and no significant difference between Group C and Group D (*P* = 0.408 > 0.05), but there was significant difference when Group A or B was compared with Group C or Group D (*P* < 0.05)

The postoperative VAS pain scores of all groups reached excellent and good levels, and the mean VAS pain scores of each group were: 1.97 ± 1.60 in Group A, 2.07 ± 1.66 in Group B, 1.197 ± 1.5265 in Group C, and 1.044 ± 1.4662 in Group D (see Fig. 2). Postoperative pain was the most-frequent chief complaint of patients. Patients in Group C and Group D had significantly less pain than those in Group A and Group B, the difference had statistical significance (*P* < 0.05). The effect sizes were medium in comparation AC, AD, BC, BD (g = 0.493, 0.592, 0.537, 0.629, g > 0.4). (Table 1) However, three female patients (1each in groups A, B and D, 1 unilateral and 2 bilateral) had severe uncontrolled pain with no obvious explanation, which lasted for more than 2 years on average. One of them (group A) had to go around in a wheelchair. Objectively speaking, her arthroplasty function recovered well and had achieved a good ROM, without any signs of infection or metal allergy, X-ray showed no signs of prosthesis in malposition or loosening. However, the pain was around the knee with the site uncertain.

#### Knee extension function and flexion function

**Fig. 3:**
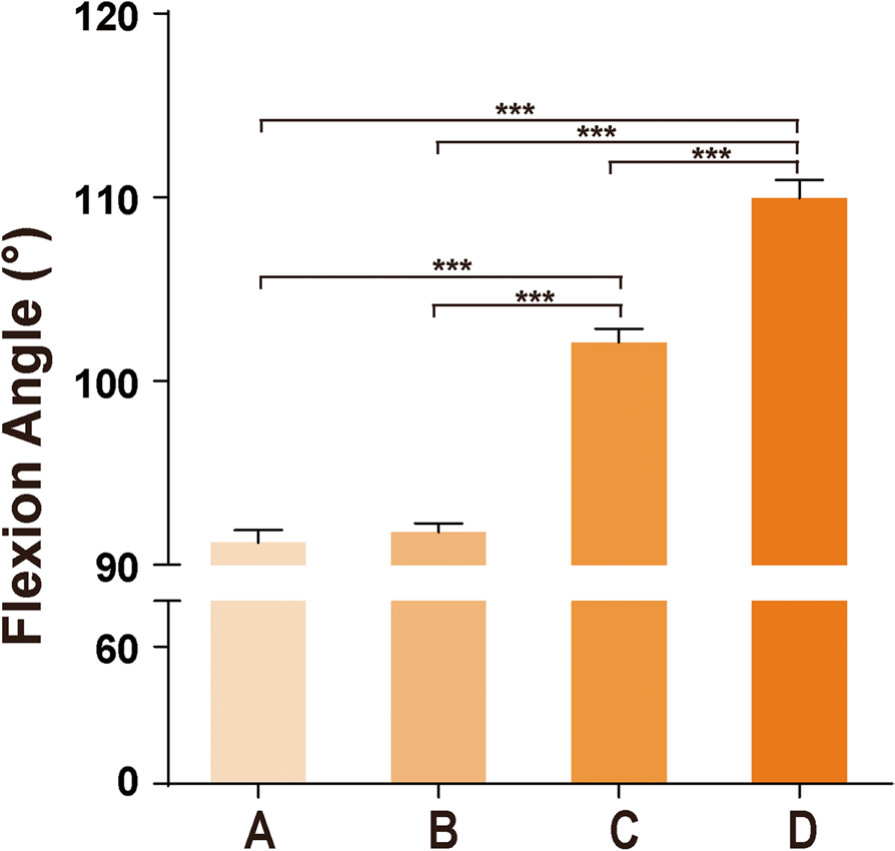
Knee flexion ROM of each group. There was no significant difference between Group A and Group B (*P* = 0.985 > 0.05), but there was significant difference when Group A was compared with Group C and Group D (*P* < 0.05); there was significant difference when Group D was compared with Group A, Group B and Group C (*P* < 0.05)

Average flexion ROM of knees in each group after surgery were over 90° (see Fig. 3): 91.21 ± 11.69° in Group A, 91.77 ± 11.95° in Group B, 102.12 ± 11.38° in Group C; 109.96 ± 10.64° in Group D; Group D is the most excellent among the four groups. There were no significant differences between Group A and Group B (*P* > 0.05), while there was significant difference when Group A and Group B was compared with Group C and Group D (*P* < 0.05) respectively; there was significant difference when Group D was compared with Group C (*P* < 0.05). The effect sizes were large in comparation AC, AD, BC, BD (g = 0.944, 1.644, 0.877, 1.547, g > 0.8). It was medium in comparation CD (g = 0.704 g > 0.4). (Table 1) There were 24 patients with flexion ROM of knee less than 90 in group A, accounting for 8.11% in this group, of which 3 patients had only 70° with difficulties in standing up and sitting, and 1 patient had reoperation to have the knee released; there were 29 (4.72%), 8 (3.46%), and 2 (1.74%) patients with poor knee function (with flexion ROM of knee no more than 90°) in Group B, C, and D, respectively, no releasing were carried out in these cases of the three groups.

**Fig. 4:**
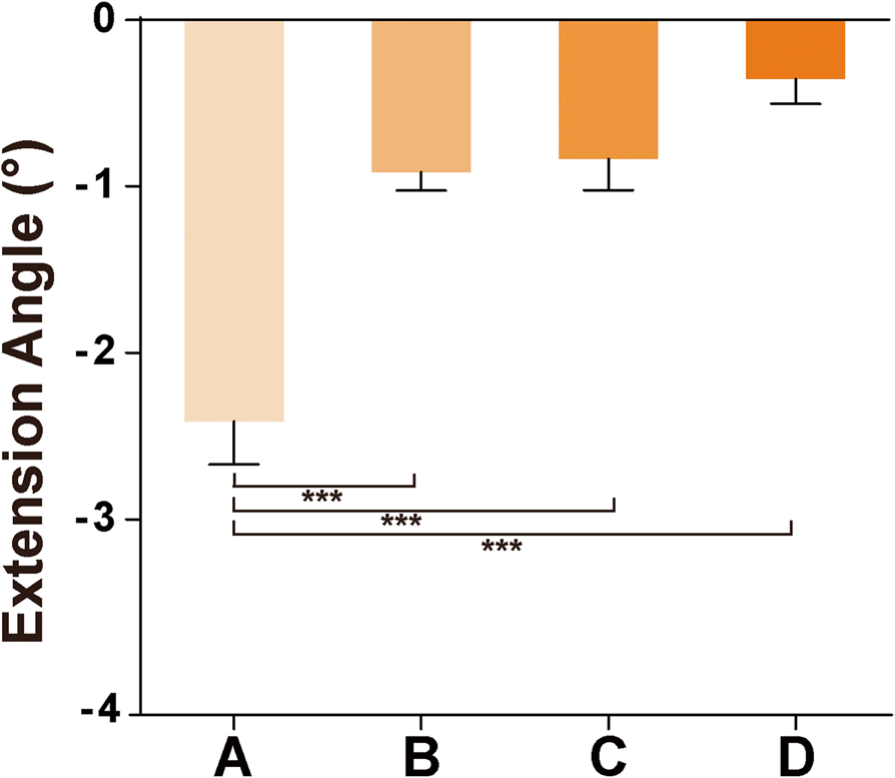
Knee joint extension ROM of each group. There was significant difference when Group D was compared with Groups A, B and C (*P* < 0.05), and there was no significant difference when Group B was compared with Group C (*P *< 0.05)

If a knee cannot be fully extended postoperatively, it will affect the knee gait. The extension ROM of knee in each group is: – 2.41 ± 4.49° in Group A, – 0.91 ± 2.82° in Group B, – 0.83 ± 2.87° in Group C, – 0.35 ± 1.60° in Group D. There was no significant difference between Group B and Group C, while there were significant differences, respectively, among Group A, Group B or C, and Group D. (Fig. 4). The effect sizes were medium in comparation AB, AC, AD (g = 0.434, 0.409, 0.527 g > 0.4). It was small in comparation BD, CD (g = 0.210, 0.190 g > 0.15). (Table 1) During the retrospective study, we found the knees of 8 patients in Group A (2.68%) unable to be completely extended but could adjust within a 3° range, there was no further surgical release in the patients; the knees of 11 patients (1.79%) in Group B could not be extended completely, and 4 patients (1.71%) in Group C, 1 patient in Group D (0.87%), none of them affected walking, and there were no cases with releasing surgery.

## Discussion

### There is significance differences among different group

White et al. [17] believes that perioperative education can well improve the postoperative knee flexion, 72% of the patients with good perioperative education can kneel with their affected knees 1 year after the surgery. Our pilot study showed that there were significant differences among these groups. The KSS scores improved as the increase of education level. The majority of patients with poor KSS scores were illiterate patients.

Though the VAS sores of all groups reached good level, the VAS sores of the patients under elementary school degree (group A and group B) had significant different between those above high school degree (group C and group D). Higher education could perform batter.

Poor function is another frequent complaint from patients. The patients in the illiterate group had the worst flexion function in this study, and the proportion of patients with flexion less than 90° in the illiterate group was 8.11%, in which 1 patient finally underwent release surgery. Scientific practices are particularly important for the recovery of postoperative knee extension and flexion function.

### Different education may have different performance in rehabilitation

The acceptance of perioperative knowledge, such as eliminating fear, avoiding rough training, controlling training methods and time, and comprehensive pain control, largely depends on the patient's education level [18].During follow-up, most patients in Group A could not fully understand the rehabilitation program and needed repeated explanations and reminding from the physiotherapists, while patients in Group D could accurately describe the postoperative rehabilitation program and exercise as requested. Many illiterate patients lived in countryside gave up active and passive rehabilitation activities due to pain. While higher education level group patients could communicate or consult with doctors through the WeChat in peaceful moods and have better medical compliances, even if dissatisfied with the outcomes, they could face complication without or less anxiety.

### Lower education level patients perform seemed not good

Many patients in the illiterate group and primary school group had poor hearing and vision to read the guide book for rehabilitation, also were not easy to remember the key points of rehabilitation training. Especially the patients aged over 70 who lived in remote areas and had great difficulties in communication, could not obtain the guidance from doctors after discharging because most of them were not capable of using smartphones. These patients were laissez-faire with fear or worry, didn’t act scientific training. Moreover, since young people (their children) worked outside, only patients themselves were at home, which led to the lack of the supervision, the effectiveness of rehabilitation for these patients was often greatly reduced [19]. And as their descriptions of the condition were unclear in smartphone interviews, we have tried a smartphone APP software, by which the range of motion can be measured and automatically sent to the APP through the induction device bound to the ankle of a patient. However, they often gave up the testing due to difficulty in using the smartphone or poor network signal, their pictures and videos for follow-up could only be obtained through other relatives and neighbors. The use rate of this APP by patients with junior high school education or above was relatively higher, but soon they ceased because of expensive medical costs.

### Higher education level patients perform seemed better

Patients with high school education or above were good at comprehending the goals in rehabilitation, and their compliance were significantly higher than that of other groups. While most patients in high school education and above group lived in cities, accompanied by their young children, the postoperative rehabilitation of those patients was effective, their follow-ups were more regular than other groups, only 2 patients with poor function (which accounted for 1.74%, it is far lower than that in the other three groups). Education level also shows advantages in postoperative knee extension function, optimizing pre-operative TKR education were helpful to improve outcomes of total knee arthroplasty.

### Education level is an independent factor affecting the rehabilitation of TAK

Enhanced rehabilitation programs (ERPs) was developed based on the ERP principles presented by Kehlet [20], which normally include in-depth preoperative patient education (PPE) [21]. PPE received by patients can effectively increase WOMAC score and Eq-5D-5L Health Status Index 1 year after knee and hip arthroplasty [12, 22]. The more knowledge of surgical procedures and rehabilitation procedures the patients get, the better their rehabilitation outcome the patients would achieve after discharge [6]. However, the degree of knowledge and information that patients with total knee arthroplasty or total hip arthroplasty obtain from a PPE session partly depends on the self-education level of the patients.

It is a common sense that education level is an independent affection factor for self-efficacy in rehabilitation after total knee arthroplasty [23]. Even some researchers hold negative views, Sun et al. [24] divided patients of tumor into two groups based on education level: junior high school group and high school above group, it was found that education level was not an influencing factor on function after tumor-type prosthesis replacement in patients (only 20 Cases) with knee osteosarcoma. Ding et al. [25, 26] analyzed the education levels of 96 patients with unilateral total knee arthroplasty and revealed that education level was not an independent risk factor, but medical compliance, which was closely related to education level, was an independent risk factor. It was also confirmed by A’bulaiti et al. [12] that treatment compliance, which was closely related to education level, was an independent risk factor affecting joint function, and the medical compliance was as high as 86.59% in the highly-educated group (HSS score ≥ 70). Yao et al. [10] grouped total knee arthroplasty patients based on two educational levels: below junior high school (31 Cases) and above high school (64 Cases), and they found out a significant difference between the two groups in self-efficacy scores (*P* < 0.05), which demonstrated that education level is an independent risk factor affecting the rehabilitation after knee arthroplasty.

Different classification of education level maybe the cause of this contradictory, diversified education level classifications appeared in recent literatures [27]. Cavanaugh et al. [17] divided the education level of patients into four groups: less than high school, high school diploma or general educational development, some college or vocational training, and baccalaureate or about, in his study, there were no elementary group and illiterate group, and only 2.9% of the people with low education, which was quite different from our grouping for this study. Anderson [3] divided the patients into multiple levels in combination with local patients’ education: illiterate, undergraduate degree, postgraduate degree and so on, the number of patients with university and postgraduate education in this study is too small to be suitable for grouping. Pua et al. [28] divided patients into four groups according to local education status: illiteracy, primary education level, secondary education level, and tertiary education level, when the knee pain and walking limitations of patients 6 months after surgery were observed, it was found that differences occurred under this classification. In our study nearly half of the patients were only primary school education, and more than 1/5 of the patients were illiterate. These proportions were much greater than that reported in the literatures [3, 17, 28, 29]. While the junior high school patients have received a complete 9-year compulsory education, it is meaningful to be set as a group alone. In this study, the patients were divided into four groups: illiterate, elementary school, junior high school and high school above level, which was in line with the educational status in western China in the 1960s[6] and was a more reasonable classification. And there were much more female patients totally, accounting for 81.9%, which were similar to literature reports, these patients were generally over 65 years and lived in countryside.

### *Education level could also affect the rehabilitation *via* patient's psychology*

Postoperative pain is the most common symptom complained by patients [29]. The preoperative patient's psychology is an important factor affecting the pain of patients after total knee arthroplasty [18, 30]. It is still a difficulty that how to identify better whether patients are at high risk of complications owing to psychiatric disorders [13]. Kong et al. [23] clarified that education level was an independent risk factor for acute postoperative pain in total knee arthroplasty patients (0R = 1.23). The study by Pua et al. [28] found that education level would affect postoperative pain, and pain relief was significantly better in patients with higher education level, but there was no statistically significant difference. Preoperative and postoperative early intervention of depression psychology could play an important role in improving postoperative pain, whether education level was associated with negative psychological disorders was not further elaborated [31]. In this study, we found that patients with education level above junior high school had significantly more pain relief and less anxiety than patients at primary school and illiterate groups. The degree of pain relief in the junior high school group and that in the senior high school above group was very similar, there was no difference between them, both were better than that in previous two groups, and the difference between them was significantly higher 3 months after surgery. In this study, there were 3 female patients of unexplained postoperative intractable pain, without evidence of infection, prosthesis loosening, prosthesis malposition, etc. Except for the junior high school group, there was 1 of the 3 female patients in each of the other three groups. This intractable pain did not seem to be related to the education level, but illiterate patients showed more severe depressive symptoms and repeatedly worried about future life and other matters. This study believes that education level is one of the influencing factors. However, more research is needed to determine whether postoperative pain can be predicted from education level [28].

### Limitations of the study

There are also limitations in this study. The psychological anticipation and psychological status of patients in the studies were not analyzed in depth before surgery, including whether the accuracy and precision in their describing the degree of pain were consistent.

## Conclusions

The rehabilitation of total knee arthroplasty is an important factor that affects the happiness of the patients. Higher education level has positive effect when it comes to the rehabilitation of the surgery.

## Data Availability

The data sets used and analyzed during the current study are available from the corresponding author on reasonable request.
